# *Sodium New Houttuyfonate*: a comprehensive review of pharmacological advances in antimicrobial, antitumor, and adjuvanticity effects

**DOI:** 10.3389/fphar.2026.1799614

**Published:** 2026-03-26

**Authors:** Lihua Fang, Dexin Yang, Jie Ning

**Affiliations:** 1 Department of Endocrinology, Shenzhen Longhua District Central Hospital, Shenzhen, China; 2 Department of Medical Laboratory, Shenzhen Longhua District Central Hospital, Shenzhen, China

**Keywords:** antimicrobial resistance, antitumor, drug repurposing, immunoadjuvant, *Sodium New Houttuyfonate*

## Abstract

*Sodium New Houttuyfonate* (SNH) is a structurally modified derivative of *Houttuynia cordata Thunb.*, emerging as a promising multi-target agent with potential applications in antimicrobial, antitumor, and adjuvant therapies. Here, we present the first review comprehensively summarizing recent pharmacological advances of SNH, with a focus on its broad-spectrum bioactivities and therapeutic repurposing potential. Literature up to December 2025 was retrieved from PubMed, Web of Science, Scopus, and CNKI, prioritizing experimental studies on SNH’s synthesis, detection, and biological functions. SNH exhibits potent antibacterial and antifungal effects against drug-resistant pathogens through mechanisms involving membrane disruption, quorum-sensing inhibition, and oxidative stress induction. Moreover, it demonstrates antitumor activity in lung, breast, and liver cancer models by regulating non-coding RNA networks, inducing apoptosis and pyroptosis, and provoking oxidative damage. Notably, SNH also acts as an immunoadjuvant, enhancing both cellular and humoral immune responses in vaccination settings. Although the preclinical evidence is compelling, further research is needed to clarify its mechanisms, pharmacokinetics, and safety *in vivo*. This review underscores the multifaceted potential of SNH and highlights the importance of translational efforts toward its clinical development against resistant infections, cancer, and as a vaccine component.

## 1 Introduction

**FIGURE 1:**
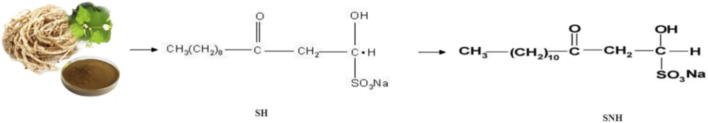
The chemical structure of *Sodium Houttuyfonate* (SH), SNH (CAS: 1847-58-1). Mw = 331.4233 g/mol.

The emergence of multidrug-resistant bacteria (Zou H. et al., 2025) and fungi (Zhou H. et al., 2025), coupled with a lack of novel adjuvants for bacterial vaccines (Mohan et al., 2013), constitutes a growing global public health threat and underscores the urgent need for developing new therapeutic agents. These antibiotic-resistant pathogens, driven by horizontally acquired resistance determinants, pose a critical challenge that has intensified the search for phytomedicinal strategies, particularly those leveraging the synergistic potential of plant-derived compounds with conventional antibiotics (Lawani et al., 2024). And the complexity of the tumor microenvironment highlights the need for therapeutic agents that address the limitations of traditional single-target paradigms (Zou Y. et al., 2025). In this context, the development of effective pharmacological agents remains a critical and ongoing priority. Considering that the long and costly process of developing entirely new molecular entities may exceed a decade, turning to natural products with a history of alternative therapeutic use represents a viable and efficient strategy, necessitating comprehensive research into their pharmacological activities. Natural products, especially those derived from traditional medicines, have long served as invaluable sources for drug discovery (Moloney, 2016). Such as artemisinin, isolated from *Artemisia annua* for treating malaria (Zou M. et al., 2025), a discovery that earned Youyou Tu the Nobel Prize in Physiology or Medicine (Su and Miller, 2015). Following this meaningful paradigm of translating traditional medicine into modern therapy, *Sodium New Houttuyfonate* (SNH), a structurally optimized derivative of the bioactive component from *Houttuynia cordata Thunb*., exhibits a diverse and potent bioactivity profile (Figure 1). Recent studies reveal that SNH possesses broad-spectrum antibacterial and antifungal effects, promising antitumor activity, and notable immunomodulatory functions, including adjuvant properties. Here we summarize the recent pharmacologic advances of SNH, which may illuminate its potential for development and repurposing in combating drug-resistant infections, cancers, and improving vaccine efficacy.

## 2 Literature search methods

**FIGURE 2:**
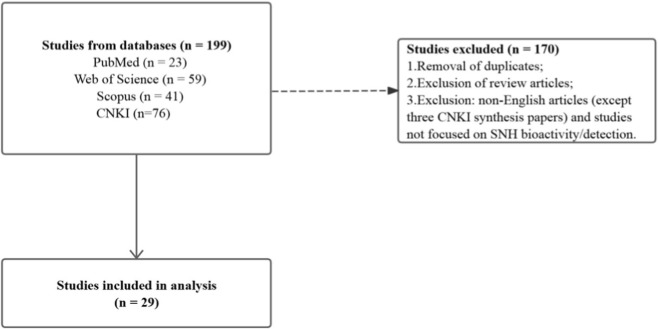
Flow diagram of study selection. Of 199 records identified, 170 were excluded due to duplicates, review articles, or irrelevant studies (non-English except three CNKI synthesis papers; not focused on SNH bioactivity/detection), leaving 29 studies for analysis.

Given the scarcity of literature on SNH, we conducted searches using the full name of *Sodium New Houttuyfonate* as the key term across PubMed, Web of Science, Scopus, and CNKI, with the timeframe set until December 2025 (Figure 2). Articles were selected based on the following inclusion criteria: (1) focus on the pharmacological functions of SNH, (2) published in English, and (3) experimental assessment of SNH’s chemical synthesis, detection and bioactivity (e.g., antibacterial, antifungal, antitumor, or immunomodulatory effects). Exclusion criteria were: (1) review articles, (2) studies primarily concerning *Sodium Houttuyfonate* (SH), and (3) articles unrelated to SNH’s biological activity or that in Chinese. After applying these criteria, three Chinese papers on chemical synthesis from CNKI, two English publications on SNH detection, and twenty-four English experimental studies on the pharmacological functions of SNH were included for analysis.

## 3 Chemical synthesis of SNH

*Houttuynia cordata Thunb* (Figure 1). has been traditionally used for treating inflammatory and infectious diseases, with *houttuynin* (*decanoyl acetaldehyde*) identified as a key volatile bioactive component (Laldinsangi, 2022). Nevertheless, the clinical application of *houttuynin* is limited by its chemical instability. To address this, *Sodium Houttuyfonate* (SH) was developed as a stable adduct formed between houttuynin and sodium bisulfite (Zhu et al., 2015). Further structural optimization, involving elongation of the aliphatic chain, led to the synthesis of SNH (Yang et al., 2007), a derivative with improved lipophilicity and stability designed for enhanced pharmacological performance. Nonetheless, SH itself exhibits limited lipophilicity (Chen et al., 2014), prompting further structural optimization through elongation of the aliphatic chain from the original decanoyl (C10) to a dodecanoyl (C12) chain, yielding SNH (Yang et al., 2025). This two-carbon extension increases the hydrophobic surface area of the molecule, enhancing its ability to interact with and insert into lipid bilayers, thereby improving membrane permeability and overall lipophilicity. The resultant derivative demonstrates both enhanced stability and optimized pharmacological performance.

**FIGURE 3:**
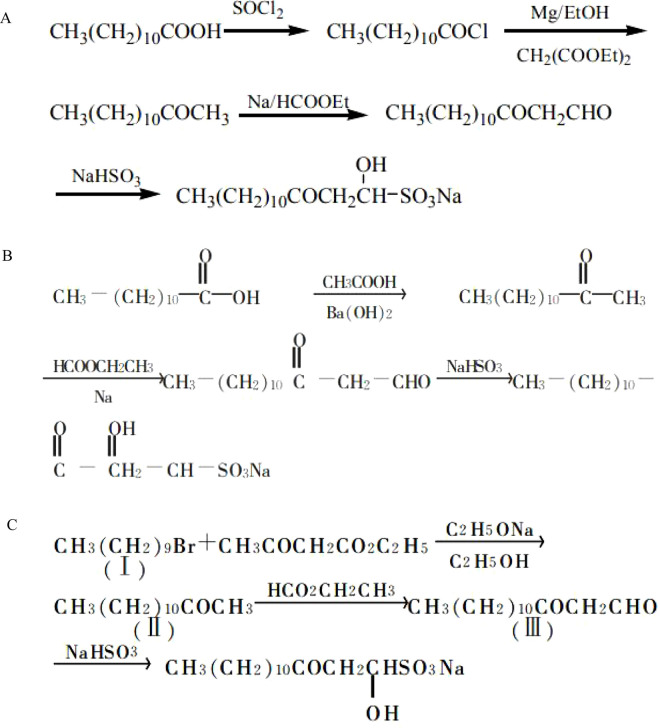
The different synthetic route of SNH. Route **(A)** Lauric acid-based sequential synthesis; Route **(B)** Barium salt pyrolysis approach; Route **(C)** Ethyl acetoacetate alkylation route.

To meet the demands of large-scale production, several efficient chemical synthesis routes for SNH have been established, moving beyond reliance on plant extraction. These synthetic strategies commonly target the construction of the core α-hydroxy sulfonate structure appended with a dodecanoyl (C12) chain, reflecting SNH’s definition as the dodecanoyl analogue. One widely adopted approach starts from readily available dodecanoic acid (lauric acid). This route typically involves conversion to the acid chloride, followed by acylation with diethyl malonate. Subsequent decarboxylation yields the key intermediate 2-tridecanone. This ketone then undergoes a Claisen condensation with ethyl formate to generate dodecanoyl acetaldehyde, which is finally trapped by sodium bisulfite to furnish SNH. This multi-step sequence has been optimized for industrial feasibility, reporting total yields of approximately 52%–56% (Chen et al., 2009) (Figure 3A).

Alternative synthetic pathways have also been explored (Figure 3B). One method utilizes a barium salt pyrolysis process, where a mixture of barium dodecanoate and barium acetate is thermally decarboxylated to produce methyl undecyl ketone, which is then elaborated to SNH (Zhu et al, 2004). Another study details an improved synthetic route for SNH, also referred to as sodium lauroyl-α-hydroxyethyl sulfonate (Figure 3C). The synthesis is a three-step process starting from readily available and cost-effective raw materials. The first step involves the alkylation of ethyl acetoacetate with 1-bromodecane, followed by decarboxylation, to yield 2-tridecanone with a yield of 75.8%. In the second step, this ketone intermediate undergoes a Claisen condensation with ethyl formate to produce dodecanoyl acetaldehyde (lauroylacetaldehyde) in a 72.8% yield. The final step is the nucleophilic addition of sodium bisulfite to the aldehyde group of dodecanoyl acetaldehyde, forming the target compound SNH with a high yield of 94.5%. The overall yield for the complete sequence is reported as 52% (calculated from 2-tridecanone). The method operates under mild reaction conditions, utilizes accessible starting materials, achieves a stable and satisfactory overall yield, and is well-suited for industrial-scale production (Zhang and Zhang, 2006).

## 4 The detection of SNH

SNH is characterized as a white or off-white crystalline powder with a slight characteristic odor, which becomes distinctly fishy upon exposure to water (Yang et al., 2016). Its solubility profile indicates ready solubility in hot water, slight solubility in water or ethanol, and near insolubility in chloroform or benzene. Additionally, SNH is soluble in sodium hydroxide (NaOH) solution, where it undergoes decomposition to regenerate the parent aldehyde and free sulfite. From an analytical perspective, one study (Yang et al., 2007) established a highly sensitive flow injection chemiluminescence (CL) method for quantifying SNH. This method capitalizes on the synergistic enhancement effect of Tween 80 and rhodamine B in an acidic medium, achieving a notably low detection limit and demonstrating utility for quality control of pharmaceutical injections. Nevertheless, the proposed applicability to biological fluid analysis is substantially limited by an inadequate assessment of potential matrix interferences, particularly in complex media such as serum. The suggested CL mechanism is based on energy transfer from excited sulfur dioxide to rhodamine B. The sulfur dioxide itself comes from oxidizing a compound known as SNH or from bisulfite. What exactly Tween 80 does in this process is still unclear and requires more research.

Recently, a novel fluorogenic probe-based method was developed for the selective detection of SNH. The probe, 4-methylumbelliferyl-2,4-dinitrobenzene sulfonate (4-MUDNBS), is initially non-fluorescent. Under alkaline conditions (pH 8.9), SNH decomposes to release sulfite ions, which subsequently react with 4-MUDNBS via a nucleophilic cleavage mechanism to yield highly fluorescent 4-methylumbelliferone (4-MU), resulting in a significant increase in fluorescence intensity. This reaction is both selective and sensitive, with a linear detection range of 0.5–15 μg/mL and a limit of detection as low as 0.15 μg/mL. Structural analysis revealed that electron-withdrawing nitro groups on the benzene sulfonyl moiety of the probe markedly facilitate the cleavage reaction, thereby enhancing detection efficiency. This fluorescent probe method (Yang et al., 2010) exhibits good tolerance to common pharmaceutical excipients and metal ions, although interference from Cu^2+^, citrate, and ascorbic acid may require consideration in complex matrices. The assay has been successfully applied to synthetic SNH samples with satisfactory recovery rates, demonstrating its potential for practical use in pharmaceutical analysis.

## 5 Recent advances in SNH pharmacological activities

### 5.1 Broad-spectrum antibacterial mechanisms and resistance modulation potential

**FIGURE 4:**
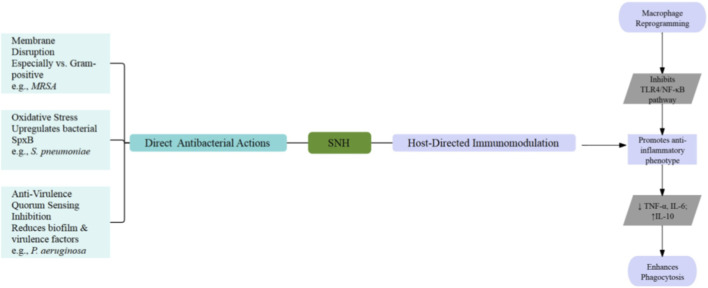
Putative antibacterial mechanisms of SNH. SNH appears to disrupt bacterial membranes, provoke oxidative stress, impede quorum sensing and biofilm formation, and influence host immune responses.

The antibacterial profile of SNH is defined by multi-target mechanisms, encompassing direct actions against bacterial cells and indirect modulation of host immune responses. The emphasis of these mechanisms varies across bacterial species, underpinning its broad-spectrum potential and synergy with conventional antibiotics (Figure 4). A primary direct action involves the disruption of bacterial membrane integrity, largely attributable to SNH’s surfactant-like molecular structure. This effect is particularly potent against Gram-positive pathogens. For instance, against methicillin-resistant *Staphylococcus aureus* (MRSA), SNH exhibits intrinsic bacteriostatic activity (MIC_50_ = 32 μg/mL) and demonstrates significant synergy with β-lactams and aminoglycosides such as oxacillin and netilmicin. Checkerboard assays reveal fractional inhibitory concentration indices as low as 0.25–0.38, indicating that sub-inhibitory concentrations of SNH drastically reduce the effective MIC of partner antibiotics. This synergy is hypothesized to result from membrane perturbation, which facilitates intracellular antibiotic accumulation. Such synergistic potentiation aligns with a broader paradigm in which phytophenolic derivatives function as ameliorative agents against microbial superbugs, enhancing the efficacy of conventional chemotherapeutics through complementary mechanisms of action and synergistic selectivity (Bayode et al., 2024). Conversely, these compelling *in vitro* findings require validation in animal infection models, and the precise molecular target of SNH remains to be definitively established (Lu et al., 2013).

Building upon its membrane-disruptive property, SNH potentiates the efficacy of berberine chloride against drug-resistant *staphylococci*, including persister cells. While berberine alone shows limited potency (MIC_90_ = 512 μg/mL), its combination with SNH reduces the MIC by 4- to 64-fold and enables bactericidal activity. Mechanistic evidence indicates that SNH increases bacterial membrane permeability, thereby promoting the intracellular accumulation of berberine. A significant limitation is that all evidence remains at the *in vitro* stage, lacking essential *in vivo* validation and comprehensive pharmacokinetic and safety profiling (Li et al., 2020). Beyond Gram-positive bacteria, SNH demonstrates targeted activity against specific Gram-negative pathogens. In *Fusobacterium nucleatum*, an anaerobe implicated in colorectal cancer progression, computational docking predictions suggest that SNH may interact with the FadA adhesin, but this proposed mechanism requires further biochemical validation, such as surface plasmon resonance (SPR) or isothermal titration calorimetry (ITC) studies, to confirm direct binding and functional inhibition (Jia et al., 2022). Transcending physical membrane damage, SNH can induce lethal metabolic disturbances within bacterial cells. Against *Streptococcus pneumoniae* (Yang et al., 2016), SNH triggers a bactericidal cascade by upregulating pyruvate oxidase (SpxB), leading to a fatal accumulation of hydrogen peroxide and oxidative stress. Proteomic analyses further revealed concomitant downregulation of proteins involved in ribosomal function and biosynthesis, indicating comprehensive cellular disruption. This mechanism of oxidative stress induction presents a distinct pathway for bacterial killing.

For Gram-negative pathogens like *P. aeruginosa*, SNH employs a potent anti-virulence strategy centered on quorum sensing (QS) inhibition. Transcriptomic analyses show significant downregulation of core QS genes (e.g., rhlI, pqsA), resulting in suppressed production of virulence factors and biofilm formation. *in vivo* efficacy was supported by improved survival in a *Galleria mellonella* infection model. In contrast, the translational potential of this approach is constrained by SNH’s high MIC (2048 μg/mL) and its primarily bacteriostatic profile against *P. aeruginosa* (Zhao et al., 2020). Complementing these direct and anti-virulence effects, SNH exhibits notable immunomodulatory properties that enhance host defenses. In macrophage models, a non-toxic concentration of SNH enhances phagocytic and bactericidal capacity while suppressing pro-inflammatory cytokine release via inhibition of the TLR4/NF-κB pathway. This host-directed mechanism suggests potential for mitigating infection-associated tissue damage. These conclusions, notwithstanding, are drawn primarily from *in vitro* cell line studies and require validation in physiological infection contexts (He X. et al., 2023).

Notably, emerging evidence suggests that the *in vivo* antibacterial efficacy of SNH may be largely mediated through indirect, host-centric mechanisms rather than potent direct killing. A pivotal study demonstrated that SNH treatment in mice significantly reshaped gut microbiota composition and upregulated key pro-inflammatory mediators. This microbial and immunological shift correlated with enhanced pathogen clearance, while SNH exhibited relatively high MIC values *in vitro*. Therefore, SNH’s antibacterial character should be contextualized within a dual framework, one aspect concerning its modest direct antimicrobial activity, and a more significant aspect involving an indirect, host-mediated strategy driven by microbiota remodeling and immune activation (Shui et al., 2021).

Crucially, emerging evidence reframes the antibacterial character of SNH beyond direct pathogen targeting, positioning it primarily as an immunomodulatory agent with indirect, host-mediated efficacy. A pivotal study demonstrated that SNH treatment in mice significantly reshaped gut microbiota composition, notably increasing the relative abundance of specific Gram-negative genera such as *Escherichia-Shigella* and *Odoribacter* (Yang et al., 2019). This microbial remodeling coincided with the upregulation of key pro-inflammatory mediators such as IFN-γ and NF-κB. It was proposed that components from these modulated bacteria stimulate the host’s mucosal immune system, thereby enhancing systemic pathogen clearance. This model is strongly supported by the congruent observation that SNH itself exhibits only mild direct antimicrobial activity *in vitro*, as evidenced by its relatively high MIC values against many pathogens. Consequently, the antibacterial profile of SNH must be contextualized within a dual framework. The first facet encompasses its modest direct activity, which includes mechanisms such as membrane disruption, oxidative stress induction, and quorum sensing inhibition. The second and potentially more significant facet involves an indirect, host-centric strategy. This strategy is driven by selective remodeling of the gut microbiota, as evidenced by the enrichment of genera including *Escherichia-Shigella* and *Odoribacter* (Yan et al., 2020), and results in the subsequent activation of host immune responses.

Conclusively, SNH orchestrates a multifaceted defense against bacterial pathogens through direct mechanisms, including membrane perturbation, oxidative stress induction, and quorum sensing interference, complemented by indirect, host-directed actions such as immunomodulation and gut microbiota remodeling (Table 1). This latter capacity is evidenced by its selective enrichment of specific bacterial genera, including *Escherichia-Shigella* and *Odoribacter*, which promotes immune activation and pathogen clearance. While this diverse mechanistic portfolio underscores its broad-spectrum potential and synergy with conventional antibiotics, the supporting evidence remains largely confined to preclinical studies. Major limitations include the need for thorough *in vivo* therapeutic evaluation and deeper elucidation of its primary molecular targets (Liu et al., 2024). Beyond this, the marked contrast between its modest direct *in vitro* activity and its significant host-mediated efficacy *in vivo* highlights the necessity for integrated translational research to precisely define its therapeutic role and advance its clinical development (Yuan et al., 2006).

**TABLE 1:** Summary of the antimicrobial and antifungal profile of SNH.

Pathogen	Type	MIC range	Key experimental finding	References
Bacteria				
*Staphylococcus aureus* (MRSA)	Gram-positive	MIC_50_ = 32 μg/mL	Potent synergy with β-lactams and aminoglycosides	Lu et al. (2013)
*Staphylococcus aureus* (persisters)	Gram-positive	-	Synergy with berberine chloride; enhances intracellular accumulation	Li et al. (2020)
*Fusobacterium nucleatum*	Gram-negative anaerobe	MIC = 200 µM	Reduces bacterial load in colorectal cancer xenograft models	Jia et al. (2022)
*Streptococcus pneumoniae*	Gram-positive	MIC/MBC = 25 µM	Rapid bactericidal activity via H_2_O_2_ induction	Yang et al. (2016)
*Pseudomonas aeruginosa*	Gram-negative	MIC = 2048 μg/mL	Anti-virulence; inhibits quorum sensing and biofilm formation	Zhao et al. (2020)
*Streptococcus mutans*	Gram-positive	MIC = 200 μg/mL	Inhibits biofilm and synergizes with chlorhexidine	Shui et al. (2021)
Fungi				
*Candida albicans*	Yeast	MIC_80_ = 256 μg/mL	Inhibits biofilm via Ras1-cAMP-Efg1 pathway; synergizes with fluconazole	Wu et al. (2020)
*Candida auris* (fluconazole-R)	Yeast	MIC = 32–128 μg/mL	Inhibits adhesion, aggregation, and biofilm; effective *in vivo*	Yang et al. (2025)
*Aspergillus fumigatus*	Mold	MIC = 50–100 μg/mL	Inhibits ergosterol synthesis; reduces fungal burden *in vivo*	Zhou et al. (2025a)
*Aspergillus flavus*	Mold	MIC_90_ = 64–128 μg/mL	Reduces fungal burden and inflammation in pulmonary model	Bao et al. (2025)

### 5.2 Antifungal efficacy: direct targeting and immune synergy

**FIGURE 5:**
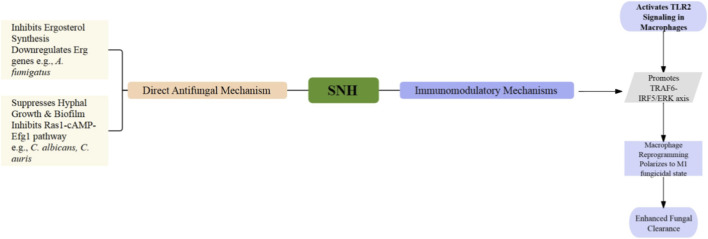
Suggested antifungal mechanisms of SNH. Proposed actions include direct suppression of ergosterol synthesis and biofilm development, alongside immunomodulatory outcomes such as macrophage polarization through TLR signaling.

The antifungal properties of SNH are mediated through a dual-pronged strategy, encompassing direct antagonism of essential fungal virulence determinants and orchestrated modulation of host immune defenses to facilitate microbial clearance (Figure 5). This multi-modal mechanism supports its potential as a broad-spectrum antifungal candidate, particularly against resistant fungal pathogens (Table 1). A principal direct mode of action involves the disruption of biofilm formation and virulence regulation in *Candida albicans* (Wu et al., 2020). SNH potently inhibits early adhesion, yeast-to-hyphal transition, and biofilm maturation, exhibiting a MIC_80_ of 256 μg/mL against strain SC5314. Transcriptomic profiling revealed that SNH downregulates core genes within the Ras1-cAMP-Efg1 pathway (e.g., ALS1, ALS3, HWP1), concurrently upregulating yeast-phase-associated markers such as YWP1 and reducing intracellular cAMP levels. It is also essential that SNH demonstrates synergistic interactions with conventional antifungals (e.g., fluconazole, caspofungin) and confers protective efficacy in a *Galleria mellonella* infection model, underscoring its capacity to subvert biofilm-associated pathogenicity.

This anti-biofilm activity extends to the emerging multidrug-resistant fungus *Candida auris*. Both SNH and its precursor SH suppress key virulence-related genes (including *Scf1*, *Bcr1*, and *EFG1*), thereby attenuating cellular adhesion, aggregation, and biofilm formation. Phenotypic analyses confirmed inhibition of biofilm development and induction of cell-wall remodeling, characterized by elevated chitin deposition. *in vivo* studies utilizing larval and murine systemic infection models demonstrated enhanced host survival, reduced fungal burden, and diminished inflammatory pathology. Nevertheless, the translational promise of this approach is tempered by the relatively high effective concentrations required and an absence of comprehensive pharmacokinetic and toxicity data (Yang et al., 2025).

For the filamentous fungus *Aspergillus fumigatus*, SNH exerts direct antifungal activity by targeting the ergosterol biosynthesis pathway. Transcriptomic and biochemical studies confirm significant downregulation of ergosterol synthesis genes (e.g., Erg2, Erg3), leading to markedly reduced membrane ergosterol content and consequent cellular disruption. Concurrently, SNH impairs critical virulence traits including conidiation, germination, and melanin production. In a murine invasive aspergillosis model, SNH administration reduced fungal load and suppressed pro-inflammatory cytokines such as IL-6 and IL-17A (Zhou X. et al., 2025). Parallel research highlights an immunomodulatory dimension of SNH’s action against *A. fumigatus*. At low concentrations, SNH binds to TLR2 on alveolar macrophages, enhancing their phagocytic and killing capacity via distinct downstream signaling axes (TLR2/TRAF6/IRF5 in uninfected cells; TLR2/TRAF6/ERK in infected cells). This effect is partly mediated through the downregulation of inhibitory microRNAs (miR-328-5p and miR-6975-3p), which amplifies antifungal immune responsiveness. On the other hand, these insights remain constrained by the lack of clinical correlation and incomplete elucidation of the underlying miRNA regulatory network (Bao et al., 2025).

Against *Aspergillus flavus*, SNH exhibits antifungal activity with a MIC_90_ ranging from 64 to 128 μg/mL, indicating moderate direct fungistatic potency. Although its mechanism is presumed to involve ergosterol pathway inhibition, based on analogous findings in *A. fumigatus,* direct mechanistic validation in *A. flavus* is lacking. In murine invasive pulmonary aspergillosis models, SNH reduced fungal burden and alleviated lung inflammation, yet potential synergistic interactions with standard antifungals remain unexplored (Zhang et al., 2022). Beyond its direct antifungal effects, SNH demonstrates prophylactic immunomodulatory efficacy in intra-abdominal *C. albicans* infection. It polarizes macrophages toward a protective M1 phenotype via the TLR2/p38/NF-κB pathway, augmenting phagocytosis and the generation of reactive oxygen and nitrogen species. This host-directed mechanism presents a complementary therapeutic strategy, though its clinical relevance is constrained by the absence of conventional susceptibility metrics (e.g., MIC values) and evaluation in immunocompromised hosts (Xie et al., 2025).

Despite the compelling multimodal antifungal profile of SNH, several material limitations curtail its translational progression. The precise molecular targets underpinning its anti-biofilm and immunomodulatory actions remain incompletely characterized, often relying on correlative omics data rather than direct mechanistic validation (Shao et al., 2017; Liu et al., 2020). Equally important is that much of the evidence derives from *in vitro* or invertebrate models (Huang et al., 2015), with a paucity of robust *in vivo* therapeutic data in mammalian systems, particularly for resistant pathogens such as *C. auris*. The effective concentrations required for direct fungistatic activity are frequently elevated, raising concerns regarding therapeutic windows and potential cytotoxicity. Although the immunomodulatory properties of SNH are promising, they have not been systematically integrated with conventional antifungals in combination therapy studies. Finally, critical gaps persist in pharmacokinetic profiling (Saint-Martin Willer et al., 2024), long-term safety assessment, and evaluation within physiologically complex host-microbe environments, each of which is imperative for advancing SNH toward clinical application in the management of fungal diseases.

### 5.3 Antitumor mechanisms: induction of multiple cell death pathways

**FIGURE 6:**
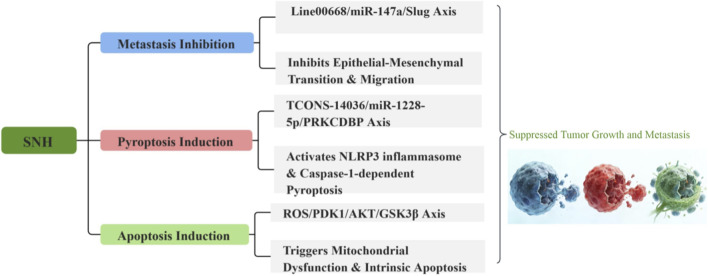
Potential antitumor mechanisms of SNH. In investigated models, SNH could modulate non-coding RNA networks, provoke oxidative stress, and trigger apoptotic, pyroptotic cell death, potentially limiting tumor progression.

The antitumor portfolio of SNH is defined by its capacity to orchestrate multifaceted antitumor effects through sophisticated molecular interventions. These actions prominently feature the strategic regulation of non-coding RNA networks, the deliberate induction of oxidative stress, and the modulation of pivotal oncogenic signaling pathways. Collectively, these mechanisms converge to suppress tumor proliferation, impede metastatic dissemination, and sensitive malignancies to adjuvant therapies, as evidenced in models of non-small cell lung cancer (NSCLC), breast cancer, and hepatocellular carcinoma (Figure 6).

In NSCLC, SNH suppresses metastatic progression by modulating a specific long non-coding RNA axis. At concentrations with an IC_50_ range of approximately 87.45–94.27 μmol/L, SNH downregulates Linc00668. This lncRNA functions as a molecular sponge for miR-147a, its downregulation by SNH consequently elevates free miR-147a levels. The increased miR-147a directly targets and suppresses the mRNA of Slug, a master transcription factor governing epithelial-mesenchymal transition (EMT), thereby inhibiting NSCLC cell migration and invasion (Jiang et al., 2019). While this elucidates a coherent RNA-mediated pathway, the study’s focus remains narrow, centered on a single molecular axis without exploring broader signaling networks or validating findings in patient-derived models.

Building upon this RNA-centric mechanism, SNH also functions as a radiosensitizer in NSCLC through the same microRNA. At an effective concentration of 0.3 mM, SNH-induced upregulation of miR-147a leads to the direct targeting and inhibition of STAT3 (Dai et al., 2021). This results in reduced STAT3 phosphorylation, attenuating its pro-survival signaling and thereby enhancing radiation-induced apoptosis in NSCLC cells. This finding suggests a promising combinatorial strategy with radiotherapy. Despite this, the evidence is confined to *in vitro* cell line models, lacking essential *in vivo* validation and a comprehensive investigation into potential off-target effects within the DNA damage response.

A distinct pro-death mechanism induced by SNH in NSCLC is the activation of pyroptosis. SNH, at concentrations of 0.1–0.4 mM, upregulates the long non-coding RNA TCONS-14036 (Jiang et al., 2023). This transcript acts as a competing endogenous RNA (ceRNA) for miR-1228-5p. By sequestering miR-1228-5p, TCONS-14036 de-represses the expression of PRKCDBP. Elevated PRKCDBP protein levels facilitate the assembly and activation of the NLRP3 inflammasome, culminating in the cleavage of gasdermin D (GSDMD) and the execution of inflammatory pyroptotic cell death. This effect was confirmed both *in vitro* and in an orthotopic xenograft model. The study’s novelty lies in linking SNH to pyroptosis, yet the precise functional role of PRKCDBP remains incompletely defined.

In breast cancer models, SNH exerts potent anti-proliferative effects primarily through the induction of catastrophic oxidative stress. It exhibits cytotoxicity with IC_50_ values of approximately 91.38 μM in MCF-7 cells and 84.48 μM in CMT-1211 cells (He L. et al., 2023) *in vivo* administration at 20 and 40 mg/kg significantly inhibited tumor growth and metastasis. Mechanistically, SNH triggers excessive accumulation of reactive oxygen species (ROS), leading to mitochondrial dysfunction. This is coupled with the inhibition of the PDK1–AKT–GSK3β signaling axis, which shifts the cellular balance toward apoptosis, evidenced by an increased Bax/Bcl-2 ratio and caspase-9 activation. A notable limitation is the use of a canine cell line for key *in vivo* modeling, which may affect direct translational relevance to human breast cancer.

An alternative, receptor-targeted mechanism has been proposed for SNH in breast cancer (Yang et al., 2023). Molecular docking analyses predict that SNH may bind to the active site of Epidermal Growth Factor Receptor Tyrosine Kinase (EGFR-TK), potentially forming interactions with key residues such as Cys773 and Asp776. While this computational prediction correlates with observed phenotypic effects in MCF-7 cells, including apoptosis induction, autophagy, and inhibited migration, direct experimental validation using binding affinity assays such as surface plasmon resonance or isothermal titration calorimetry is required to confirm EGFR-TK as a *bona fide* molecular target of SNH. Concurrently, SNH displayed considerable antinematode activity (*Caenorhabditis elegans* LC_50_ = 40.46 μg/mL), hinting at broad-spectrum bioactivity. The work remains preliminary, constrained by its reliance on computational prediction and *in vitro* correlation without direct enzymatic validation or *in vivo* pharmacokinetic behavior and safety profiles under systematic investigation.

In hepatocellular carcinoma (Yang et al., 2019), SNH demonstrates a combined pro-apoptotic and anti-metastatic profile. It shows broad-spectrum cytotoxicity, with an IC_50_ of 436.54 μg/mL for HepG2 cells. The compound induces mitochondrial pathway-mediated apoptosis, characterized by downregulation of the anti-apoptotic protein Bcl-2 and DNA fragmentation. Another critical dimension involves the suppression of cancer cell migration. SNH downregulates metastasis-associated genes, including MMP9 and VEGF, with molecular docking simulations suggesting direct inhibition of MMP9. *In vivo* validation in HepG2 tumor-bearing mice demonstrated that a dose of 300 mg/kg/day significantly reduced tumor growth. The study’s limitations include a narrow *in vitro* concentration range, testing of only a single high dose *in vivo*, and a lack of normal cell cytotoxicity data.

The antitumor profile of SNH, while pharmacologically promising, is characterized by several unresolved complexities. Current mechanistic elucidation remains compartmentalized, with identified pathways such as non-coding RNA networks and oxidative stress studied in discrete models rather than as an integrated signaling landscape (Table 2). Significant translational hurdles arise from the high effective concentrations required *in vitro*, coupled with a conspicuous absence of systematic pharmacokinetic and safety evaluations, which obscures the projection of a viable therapeutic index (Yao et al., 2022). Moreover, the evidence base draws predominantly from conventional cell lines and xenografts, systems that insufficiently replicate the heterogeneity and microenvironmental dynamics of human malignancies (Tan et al., 2023). A critical oversight is the lack of investigation into SNH’s combinatory potential with established anticancer regimens (Feldman et al., 2015). Advancing this research necessitates a shift toward systems pharmacology approaches (Zheng et al., 2018), defining *in vivo* exposure response relationships, utilizing more physiologically faithful disease models, and rigorously evaluating SNH within multimodal therapeutic strategies to credibly ascertain its translational merit.

**TABLE 2:** Summary of the antitumor activity of SNH across cancer models**.**

Cancer Type	Model system	*In vitro* activity (IC50)	*In* *vivo* effect (Dose)	References
Non-small cell lung cancer (NSCLC)	A549, HCC827 cells	∼87–94 μmol/L	Metastasis inhibition *in vitro*	Jiang et al. (2019)
NSCLC	A549, HCC827 cells	0.3 mM (radiosensitizer)	Enhances radiation-induced apoptosis *in vitro*	Dai et al. (2021)
NSCLC	A549 cells; orthotopic mouse model	0.1–0.4 mM	Induces pyroptosis *in vitro* and *in vivo*	Jiang et al. (2023)
Breast cancer	MCF-7, CMT-1211 cells	∼91 μM (MCF-7), ∼84 μM (CMT-1211)	20, 40 mg/kg (i.g.) inhibits tumor growth and metastasis	He et al. (2023a)
Breast cancer	MCF-7 cells	250 μg/mL (48h, ∼68% inhibition)	Induces apoptosis and autophagy *in vitro*	Yang et al. (2023)
Hepatocellular carcinoma	HepG2, A2780, SKOV-3, MCF-7 cells	147–437 μg/mL (HepG2: 436.5 μg/mL)	300 mg/kg/day (i.g.) reduces tumor growth by 50.8%	Yang et al. (2019)

### 5.4 Immunoadjuvant properties: enhancing vaccine efficacy

**FIGURE 7:**
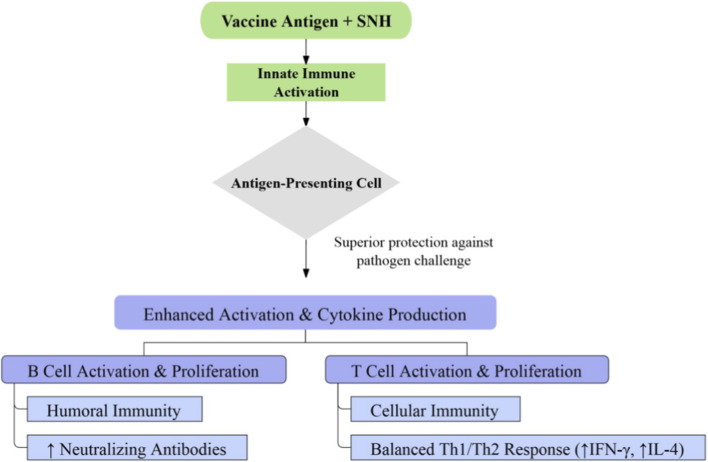
Immunomodulatory profile of SNH as a candidate adjuvant. In an avian vaccination setting, SNH was observed to enhance lymphocyte responses, elevate antibody levels, and modulate Th1/Th2 balance, supporting further evaluation in mammalian systems.

Apart from its direct antimicrobial and antitumor activities, SNH exhibits promising immunomodulatory functions that underscore its potential as a vaccine adjuvant (Figure 7). Evidence from avian models demonstrates its capacity to comprehensively enhance antigen-specific immunity, boosting both humoral and cellular arms of the adaptive immune system. In a study utilizing an avian infectious bronchitis virus vaccine, SNH was administered at concentrations ranging from 100 to 800 mg/L. The 200 mg/L dose emerged as optimally efficacious, significantly augmenting peripheral T-lymphocyte proliferation, elevating hemagglutination inhibition antibody titers, and promoting a balanced Th1/Th2 cytokine profile characterized by increased IFN-γ and IL-4 (Zhou et al., 2010). These observations collectively suggest that SNH enhances vaccine efficacy through dual enhancement of cellular and humoral immunity. Nonetheless, this foundational investigation is circumscribed by its exclusive focus on an avian species, which inherently limits extrapolation to mammalian or human immunology. A parallel requirement is the study lacks comparative evaluation against established commercial adjuvants, offers minimal insight into the molecular mechanisms governing immune potentiation, and omits assessment of formulation stability and local reactogenicity. While this foundational study provides compelling phenotypic evidence of SNH’s adjuvant activity, the underlying molecular mechanisms remain largely unexplored.

Beyond the existing avian model, forthcoming research directions for SNH as an adjuvant should encompass several critical translational dimensions. Validation in established mammalian immunization models is imperative to ascertain whether its immunopotentiating effects translate across species (Zuber et al., 2021). Head-to-head comparisons with licensed adjuvants, for example, aluminum salts (Young et al., 2015) or oil-in-water emulsions (Zhang et al., 2021), are essential to benchmark its efficacy and safety profile relative to current standards. Mechanistically, studies should delineate the cellular targets and signaling pathways through which SNH modulates antigen-presenting cell function and lymphocyte activation, which may involve specific pattern recognition receptors or cytokine networks (Zimmermann and Lepenies, 2015). Given the demonstrated role of Toll-like receptor (TLR) signaling, particularly TLR2 and TLR4, in mediating the immunomodulatory effects of SNH against fungal pathogens (Bao et al., 2025), it is plausible that its adjuvanticity may similarly involve the activation of these pattern recognition receptors on antigen-presenting cells. Future investigations should therefore explore whether SNH, akin to its action in antifungal defense, functions as a TLR agonist to enhance antigen uptake, maturation, and subsequent T-cell priming, a mechanism that would position it within the broader class of immunostimulatory phytochemical adjuvants (Bayode et al., 2024). Coupled with this is the need for comprehensive profiling of its local reactogenicity (Batista-Duharte et al., 2011), systemic toxicity, and formulation stability will be indispensable for advancing SNH toward practical vaccine development. Addressing these gaps will determine whether SNH can evolve from a promising phenotypic observation into a viable, mechanism-defined adjuvant for long-term vaccine platforms.

### 5.5 Pharmacokinetic and safety challenges

The promising preclinical efficacy of SNH notwithstanding, its clinical translation is critically hampered by the absence of rigorous preclinical pharmacokinetic and toxicity profiling. While SNH exhibits a solubility profile that may influence its oral bioavailability, systematic investigations into its absorption, distribution, metabolism, and excretion (ADME) in mammalian systems are lacking. The relatively high concentrations required for certain antitumor and antifungal effects further underscore the need to define a viable therapeutic window and assess potential off-target toxicity.

Comparative insights can be drawn from the parent compound, *sodium houttuyfonate*. Following intravenous administration in rats, SH is rapidly distributed, with the highest accumulation observed in the lungs, consistent with its traditional use for respiratory infections (Deng et al., 2012). Oral administration also leads to swift absorption into the circulatory system and multiple organs (Gao et al., 2009). Alternatively, SH bioavailability is influenced by factors such as solvent, temperature, and pH (Chen et al., 2014). Regarding safety, SH has shown a favorable profile in preclinical studies, with hemolysis below 15% at 256 μg/mL and no toxicity after 60-day high-dose administration in mice (Huang et al., 2015). However, SH can covalently bind to tissue proteins via Schiff’s base formation and activate the histamine H1 receptor, mechanisms potentially underlying adverse reactions associated with houttuynin-containing injections (Guo et al., 2014). Whether SNH, as a structurally optimized derivative, retains or mitigates these properties remains unexplored.

Future investigations must prioritize systematic ADME profiling and toxicity assessment of SNH in relevant animal models. Particular attention should be paid to potential cardiovascular effects, given that SH has been shown to inhibit voltage-gated sodium currents in cardiomyocytes (Cao et al., 2018). Elucidating these parameters will be essential for establishing a safe therapeutic index and advancing SNH toward clinical development.

## 6 Conclusion and prospect

SNH exhibits a remarkable and multifaceted pharmacological profile, positioning it as a versatile candidate for therapeutic development. Its efficacy against a broad spectrum of drug-resistant pathogens, coupled with promising antitumor activity and immunoadjuvant potential, highlights its value in addressing several major health challenges. The multi-target mechanisms of action, ranging from direct microbial membrane disruption and virulence inhibition to host immune modulation and cancer cell death induction, contribute to its potent and broad bioactivity. Even so, the current evidence primarily stems from *in vitro* and preclinical studies. Key limitations include the need for deeper mechanistic elucidation, detailed pharmacokinetic and toxicological characterization in animal models, and ultimately, validation in clinical settings. Prospective research should prioritize these aspects to bridge the gap between promising preclinical data and clinical application. Leveraging modern techniques like structure-based drug design could further optimize SNH and its derivatives. Overall, SNH represents a compelling natural product-derived lead compound with significant potential for repurposing and development into novel therapeutic strategies against infectious diseases, cancer, and as a vaccine component.
